# Hormonal regulation of metabolism—recent lessons learned from insulin and estrogen

**DOI:** 10.1042/CS20210519

**Published:** 2023-03-21

**Authors:** Zhipeng Tao, Zhiyong Cheng

**Affiliations:** 1 Cutaneous Biology Research Center, Massachusetts General Hospital, Harvard Medical School, Charlestown, Massachusetts, U.S.A.; 2 Department of Food Science and Human Nutrition, University of Florida, Gainesville, Florida, U.S.A.

**Keywords:** autophagy, estrogen, Insulin, metabolic disease, metabolism, mitochondria

## Abstract

Hormonal signaling plays key roles in tissue and metabolic homeostasis. Accumulated evidence has revealed a great deal of insulin and estrogen signaling pathways and their interplays in the regulation of mitochondrial, cellular remodeling, and macronutrient metabolism. Insulin signaling regulates nutrient and mitochondrial metabolism by targeting the IRS-PI3K-Akt-FoxOs signaling cascade and PGC1α. Estrogen signaling fine-tunes protein turnover and mitochondrial metabolism through its receptors (ERα, ERβ, and GPER). Insulin and estrogen signaling converge on Sirt1, mTOR, and PI3K in the joint regulation of autophagy and mitochondrial metabolism. Dysregulated insulin and estrogen signaling lead to metabolic diseases. This article reviews the up-to-date evidence that depicts the pathways of insulin signaling and estrogen-ER signaling in the regulation of metabolism. In addition, we discuss the cross-talk between estrogen signaling and insulin signaling via Sirt1, mTOR, and PI3K, as well as new therapeutic options such as agonists of GLP1 receptor, GIP receptor, and β3-AR. Mapping the molecular pathways of insulin signaling, estrogen signaling, and their interplays advances our understanding of metabolism and discovery of new therapeutic options for metabolic disorders.

## Introduction

Since the definition of hormone by the British physiologist Ernest Starling in 1905 [1], hormone research has advanced in many areas, including new hormone discovery, functional characterization, biotechnology-assisted synthesis, and clinical application [2,3]. A hormone was defined as a molecule produced by the glands with internal secretion and delivered by the blood circulatory system to target tissues, regulating physiological functions [1]. Nowadays, it has been recognized that cytokines produced by non-gland cells or tissues (e.g., adipose tissue, liver, and skeletal muscle) function as hormones [3–5].

Insulin is secreted from pancreatic β-cells, which is critical for metabolic health and functions of various tissues such as muscle, adipose tissue, and liver [6–9]. Studies have established canonical insulin signaling pathways (e.g., IRS-PI3K-PDK1-Akt) and non-canonical insulin signaling pathways (e.g., IRS-PI3K-PDPK1-aPKCλ) in the regulation of glucose metabolism [7,10]. Moreover, insulin signaling modulates mitochondrial metabolism including mitochondrial biogenesis, dynamics, and autophagy (mitophagy, a mitochondrial quality control system that allows for the elimination of damaged and redundant mitochondria) [11], primarily through the Forkhead box O (FoxO) transcription factors [12]. Recent research has revealed the epigenetic role of insulin signaling via Snail and Slug in metabolic disorders [13–15].

Estrogen is mainly synthesized and secreted by the ovaries and placenta, which regulates the development of the reproductive system and nonreproductive tissue (e.g., adipose tissue, liver, muscle, and brain) function and homeostasis [16]. Estrogen signaling mediates metabolic homeostasis through its receptors (especially ERα and ERβ), which modulate mitochondrial homeostasis, autophagy, and epigenetic programming [3,5,17,18]. Dysregulation of estrogen signaling leads to metabolic disorders such as obesity, diabetes, cardiovascular diseases, liver diseases, muscle diseases, and neurodegenerative diseases [16,19–22].

Accumulated evidence has linked estrogen signaling to insulin signaling in metabolic regulation. For instance, both insulin and estrogen signaling cascades are involved in the regulation of mitochondria, autophagy, and protein degradation [12,23–25]. Sirt1 and PI3K serve as the mediators in the crosstalk of insulin and estrogen signaling, which plays essential roles in metabolism [26–28]. In this article, we present an updated view of the signaling pathways of insulin and estrogen, and how they interact in the regulation of metabolic homeostasis.

## Insulin signaling and metabolism

### Insulin signaling

**Figure 1:**
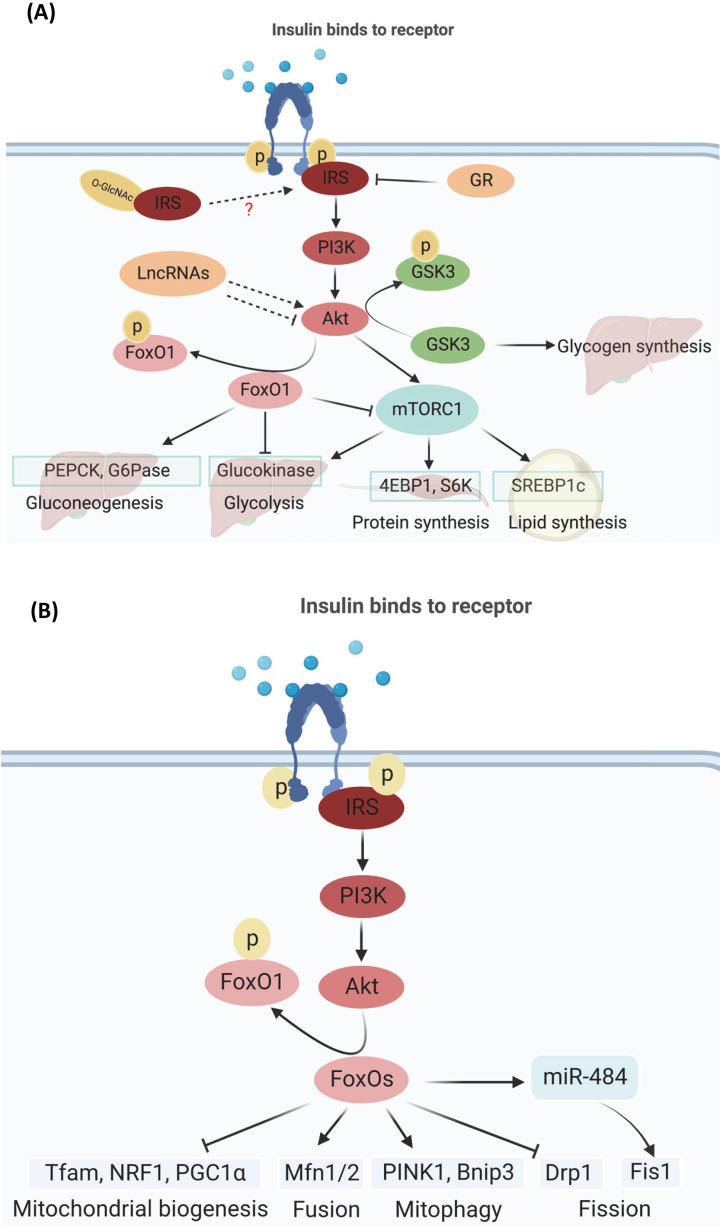
Insulin signaling in metabolism (**A**) Insulin signaling mediates macronutrients metabolism. Following canonical IRS-PI3K-PDK1-Akt insulin signaling, feeding activates IRS phosphorylation at multiple tyrosine sites and activates PI3K and Akt (phosphorylation at Thr308), which phosphorates FoxO1 (Ser256), retains it in the cytoplasm, and suppresses the expression of gluconeogenic genes, phosphoenolpyruvate carboxykinase (PEPCK) and glucose-6-phosphatase (G6Pase), while fasting promotes gluconeogenesis. Upon feeding, activated Akt suppresses glycogen synthesis via phosphorylation of GSK3 (Ser9 and Ser21). Akt induces glycolysis via FoxO1 phosphorylation and activation of glucokinase gene expression. Akt stimulates protein synthesis via mTORC1 signaling pathway. Akt activates lipid synthesis via phosphorylation of TSC and FoxO1, which activates mTORC1 and SREBP1c and the lipogenic gene. Additionally, glucocorticoid receptor (GR) transcriptionally suppresses IRS1 and promotes IRS2 expression, and IRS1 (on Ser1101) and IRS2 (on Ser1149) could be posttranslationally modified by glycosylation (O-GlcNAc), but how it interferes with phosphorylation is not known. Akt could be mediated by lncRNA, either activation or suppression. (**B**) Insulin signaling regulates mitochondrial metabolism (mitochondrial biogenesis, fusion, and fission, mitophagy). Insulin signaling mediates mitochondrial biogenesis via FoxOs-mediated Tfam, NRF1, and PGC1α. FoxOs affect mitochondrial dynamics mainly through transcriptionally up-regulate the fusion proteins Mfn1/2 and down-regulate fission protein Drp1. Additionally, FoxOs post-transcriptionally suppress mitochondrial fission via promoting microRNA-484 (miR-484) expression and its binding with Fis1 mRNA and suppresses Fis1 translation. FoxOs promote mitophagy through transcriptional regulation of PINK1 and BNIP3.

The canonical insulin signaling includes insulin receptor (IR)-mediated phosphorylation of IRSs proteins and activates phosphatidylinositol-3-kinase (PI3K) and downstream protein kinases, such as Akt (Figure 1A) [29]. During feeding status, Akt phosphorates FoxO1, retaining FoxO1 in the cytoplasm and suppressing hepatic gluconeogenesis. FoxO1 confers insulin sensitivity onto glucose-6-phosphatase expression) (Figure 1A) [10]. During fasting, IRS-PI3K-PDK1-Akt signaling was deactivated and leads to nuclear translocation of FoxO1, up-regulating gluconeogenic genes that encode phosphoenolpyruvate carboxykinase (PEPCK) and glucose-6-phosphatase, catalytic subunit (G6Pase), by coordinating with a complex of cAMP response element-binding protein (CREB) and transcription coactivator 2 (CRTC2), and CREB-binding protein (CBP) (Figure 1A) [10]. In addition, Akt phosphorylates mTORC1 to promote lipogenesis, protein synthesis, and gluconeogenesis [27,30–32] (Figure 1A). The non-canonical insulin signaling may bypass the canonical IRS-PI3K-PDK1-Akt cascade via PDPK1-aPKCλ branch [10,33]. In recent years, new mediators of insulin signaling have been discovered. For instance, insulin signaling may undergo transcriptional regulation by glucocorticoid receptor (GR) that binds to the IRS promotor (Figure 1A); interestingly, GR represses IRS1 but induces IRS2 expression [34]. At post-translational level, IRS could be glycosylated, O-GlcNAc modification of IRS contributes to insulin resistance, but how it interplays with IRS phosphorylation needs further exploration [35]. Akt as one of the key mediator of insulin signaling is under the regulation of lncRNAs, with ceRNA to activate Akt expression and other lncRNAs to down-regulate Akt (Figure 1A) [36,37].

### Insulin in the regulation of macronutrient metabolism

The homeostasis of macronutrients, such as gluconeogenesis, glycolysis, protein synthesis and breakdown, lipogenesis, and lipolysis, is regulated by insulin. Insulin level and sensitivity are affected by the feeding and fasting cycle or diseased state, which affects the metabolism of these macronutrients as well as tissue-specific and systemic functions. As insulin sensitive tissues, liver, muscle, and adipose tissue respond to insulin signaling and affect their macronutrient metabolism during feeding-fasting cycle and pathological status. In the liver, the level of insulin increased upon feeding, which signals the liver to regulate hepatic glucose, protein, and lipid metabolism [38]. Following canonical IRS-PI3K-PDK1-Akt insulin signaling, feeding activates Akt, which phosphorates FoxO1, retains it in the cytoplasm and suppresses gluconeogenic genes, phosphoenolpyruvate carboxykinase (PEPCK) and glucose-6-phosphatase (G6Pase) expressions and hepatic gluconeogenesis (Figure 1A) [10,39]. On the other hand, fasting suppressed canonical IRS-PI3K-PDK1-Akt insulin signaling and deactivated Akt-reduced phosphorylation of FoxO1, which leads to nuclear translocation of FoxO1, and FoxO1 upregulates PEPCK and G6Pase, by coordinating with a complex of cAMP response element-binding protein (CREB) and transcription coactivator 2 (CRTC2), and CREB-binding protein (CBP) [10]. In addition, upon feeding mTORC2 phosphorylates Akt on Ser473 and activates Akt to control other metabolic activities in the liver, including glycogen production, glycolysis, and lipid synthesis (Figure 1A) [38]. Akt suppresses glycogen synthesis via phosphorylation of GSK3 and GSK3 independent pathway [40,41]. In addition, Akt induces glycolysis via FoxO1 phosphorylation and activation of glucokinase gene expression (Figure 1A) [42]. Through phosphorylating and inhibiting the TSC proteins, Akt stimulates the mTORC1 and protein synthesis pathways (Figure 1A) [27,31]. Akt activates lipid synthesis through phosphorylation of TSC and FoxO1, which activates mTORC1 and SREBP1c and the lipogenic gene program since SREBP1c is a transcription factor essential for the synthesis of fatty acids, triglycerides and cholesterol (Figure 1A) [43,44].

In the feeding state, insulin drives muscle growth and muscle mass maintenance. Insulin binds to the insulin receptor (IR) and activates the IRS-PI3K-Akt pathway, which inhibits the nuclear translocalization and transcriptional activity of FoxO1, FoxO3, and FoxO4. Proteasomal and autophagy–lysosomal protein breakdown is suppressed when FoxO1, FoxO3, and FoxO4 are inhibited [45]. Furthermore, amino acids and IRS-PI3K-Akt pathway stimulate the mammalian target of rapamycin complex 1 (mTORC1) to increase protein synthesis (Figure 1A), resulting in net protein gain and muscle growth [46–48]. While fasting or diabetes reduces insulin signaling, which increases FoxO isoform nuclear translocation and transcription of critical mediators of the ubiquitin–proteasome and autophagy–lysosome systems, resulting in a marked increase in protein degradation that outweighs protein synthesis, resulting in muscle atrophy and a high-protein-turnover state [46,49]. In addition, insulin activates the group I p21-activated kinase (PAK) isoforms PAK1 and PAK2, and PAK1/2 signaling was impaired by insulin resistance in skeletal muscle [50,51]. PAK1 is essential for insulin-stimulated GLUT4 translocation in mouse skeletal muscle. PAK2 is required for insulin-stimulated glucose uptake in glycolytic extensor digitorum longus muscle, but PAK1 is not required for whole-body glucose homeostasis and insulin-stimulated muscle glucose uptake [50]. Prolonged insulin exposure induced insulin resistance in primary human skeletal muscle-derived cells (HMDCs), as characterized by blunted IRS-1 phosphorylation (Tyr612) and Akt (Ser473) phosphorylation in response to an acute insulin stimulation. Prolonged insulin exposure suppressed glucose uptake, while increased compensatory expression of glucose transporter 1 (GLUT1) [52]. As insulin insensitive tissue, brain responds to insulin signaling and regulates cell plasticity and memory. Insulin signaling activates IRS1-PI3K-PDK1-Akt-AMPK and IRS1-PI3K-c-Raf-MEK signaling, and converges on ERK and memory through RSK/CREB/CBP-dependent gene transcription, which are essential for cell plasticity and memory in the hippocampus [53,54]. At the same time, insulin signaling axis affects mediators of glucose utilization (GLUT, GSK3), mitochondrial function (FoxO1), and energy metabolism (mTOR, AMPK) to support hippocampal integrity. Thus, insulin signaling affects macronutrients metabolism through targeting FoxOs and mTOR (Figure 1A).

### Insulin in the regulation of mitochondria

Mitochondrion is an organelle that primes biochemical processes of respiration and energy production [55–57]. Its homeostasis is jointly maintained by mitochondrial biogenesis, mitochondrial dynamics (fusion or fission), and mitophagy (mitochondrial autophagy) [12,58]. Insulin signaling mediates mitochondrial function and disease status [56,57], which is fine-tuned through FoxOs and PGC1α (Figure 1B). FoxOs play key roles in the triad of mitochondrial biogenesis, dynamics, and mitophagy as a transcription factor in the canonical IRS-PI3K-Akt insulin signaling pathway [12]. FoxOs differentially regulate mitochondrial biogenesis via Tfam, NRF1, and PGC1α in different tissues (Figure 1B) [12,59,60]. FoxOs affect mitochondrial dynamics mainly through transcriptionally up-regulate the fusion proteins Mfn1/2 and down-regulate fission protein Drp1 (Figure 1B) [12,61]. Additionally, FoxOs posttranscriptionally suppress mitochondrial fission via promoting microRNA-484 (miR-484) expression and its binding with Fis1 mRNA and suppresses Fis1 translation (Figure 1B) [62]. In addition, fasting or insulin resistance promote FoxOs activity and its up-regulation on mitophagy (Figure 1B) [11]. Mechanistically, FoxOs promote mitophagy through transcriptional regulation of PINK1 [63,64] and BNIP3 (Figure 1B) [65,66].

Independent of FoxOs, insulin could mediate mitochondrial biogenesis through Akt-PGC1α and CREB- PGC1α signaling [67]. In response to insulin, activated Akt induces the phosphorylation of PGC1α by Cdc2-like kinase 2 (Clk2) and suppress mitochondrial biogenesis [68,69]. Furthermore, Akt phosphorylates CBP/P300 and suppresses the recruitment of the CREB transcription factor to induce PGC1α transcription [70,71]. Thus, insulin signaling regulates mitochondrial metabolism through targeting FoxOs-mediated mitochondrial biogenesis, dynamics and mitophagy as well as PGC1α-dependent, FoxOs-independent mitochondrial biogenesis.

### Impaired insulin signaling and metabolic diseases

Aberrant insulin signaling is most prominent in diabetes and associated metabolic diseases (e.g., diabetes and obesity). Diabetes is characterized by hyperglycemia, which is defined as a fasting blood sugar level more than 126 mg/dL [72]. Type 1 and type 2 diabetes are the two main types of the disease, which affects more than 537 million adults worldwide in 2021 [73]. Type 1 diabetes mellitus (T1DM) is an autoimmune disease in which the immune system destroys insulin-producing β cells [74]. Type 2 diabetes mellitus (T2DM) is characterized by high insulin concentrations, at least at its early stages due to insulin resistance, which contrasted the lack of insulin in T1DM [75]. Both diabetic conditions will lead to dysfunctional insulin signaling, such as IRS→PI3K→FoxO1, which would lead to metabolic dysfunctions and affect protein catabolism, lipolysis and the formation of ketone bodies [76], and cause metabolic syndrome [77,78].

Obesity and diabetes are significantly associated with the development of nonalcoholic fatty liver disease (NAFLD), especially when the patients are treated with insulin [79–81]. Clinical cross-sectional study of patients with Type 2 diabetes mellitus indicated that lower fasting and postprandial glucagon-to-insulin ratio was significantly associated with the presence of NAFLD [82]. The production of glucose and triglycerides is elevated in Type 2 diabetes and nonalcoholic fatty liver disease (NAFLD), which was supported by the selective hepatic insulin resistance theory, in which insulin drives *de novo* lipogenesis (DNL) without suppressing glucose production [83,84], In overweight human, overfeeding saturated fat increased the greatest level of increased intrahepatic triglyceride (IHTG), insulin resistance, and harmful ceramide [85], And overfeeding sugar increased *de novo* lipogenesis, while overfeeding saturated fat increased lipolysis and unsaturated fat decreased lipolysis [85]. While weight loss reduced intrahepatic triglyceride IHTG content via lowering hepatic DNL, at least in part [84]. While a clinical study on obese subjects with or without NAFLD found that NAFLD individuals attenuated, not increased, glucose-stimulated/high-insulin lipogenesis [86]. Thus, early detection and treatment of aberrant insulin signaling are critical for prevention and treatment of liver disease.

High fat diet induced obesity leads to impaired insulin access to skeletal muscle and glucose uptake, while polyunsaturated fat-rich diets improve insulin sensitivity and lower the risk of Type 2 diabetes. In comparison with a saturated high fat diet, a polyunsaturated high fat diet sustained insulin sensitivity and insulin availability to muscle [87]. In addition, recent study indicates the pathological role of myokines generated by diseased striated muscle, such as myokine/cardiokine MG53, might increase systemic insulin resistance [88,89]. Thus, it would be critical for mitigating aberrant insulin signaling induced muscle dysfunction. Correction of poor glycemic control and use of insulin leads to increased skeletal muscle mass in Japanese patients with Type 2 diabetes [90,91]. Insulin therapy for Type 2 diabetes enhanced skeletal muscle index (SMI) and protected against Sarcopenia, a loss of skeletal muscle mass and strength that occurs with age and is a primary cause of disability and mobility limits [91]. Interestingly, short-term immobilization boosts intramyocellular diacylglycerol and decreases insulin sensitivity in muscle through increased lipin1 activity [92]. Therefore, insulin plus physical activity therapy would provide promising strategies for diabetic muscle disease.

Unlike insulin-sensitive tissues (e.g., the liver, muscle, and adipose tissue), insulin does not increase glucose uptake/metabolism in the brain [93]. However, insulin regulates brain functions and neurodegenerative diseases across different regions and cross-talk with other tissues, such as liver, to fine-tune whole body function [93]. For example, insulin modulates cerebral bioenergetics, boosts synaptic survival, and dendritic spine formation and raises the turnover of neurotransmitters like dopamine, according to new findings from human and animal studies. Insulin also affects proteostasis, impacting amyloid peptide clearance and tau phosphorylation [94–97], both of which are hallmarks of Alzheimer’s disease. Insulin affects vasoreactivity, lipid metabolism, and inflammation, all of which affect vascular function. Insulin dysregulation may contribute to neurodegeneration via these numerous routes [98]. Insulin signaling has been found to be desensitized in the brains of patients, drugs that can resensitize insulin signaling have been tested to evaluate if this strategy can alter disease progression [54]. Insulin administration has shown good effects in preclinical studies [99,100]. In a 12-month double-blind clinical research of patients with amnestic moderate cognitive impairment or Alzheimer’s disease, intranasal insulin therapy had no cognitive or functional advantages in the primary intention-to-treat group [101], the limitations would be the feasibility challenge with insulin administration device and it has never been used in patients with Alzheimer’s disease before. Altogether insulin plays important roles in maintaining the physiological homeostasis in different tissues and derangement of insulin signaling contributes to diabetes, obesity, muscle diseases, liver diseases, and neurodegenerative diseases via targeting specific tissue and cross-talk among different tissues.

### New therapeutic options

Insulin and insulin analogues have been widely used for therapeutic options over the years [102]. However, the reported side effects and therapeutic inefficacy promote the development of new therapeutic alternatives, such as glucagon-like peptide-1 (GLP1) receptor agonists and glucose-dependent insulinotropic polypeptide (GIP) receptor agonists, to restore insulin secretion for the treatment of T2DM [103]. In 2017, a dual GLP1/GIP receptor agonist, NNC0090-2746, was developed and showed significant improvement of glycemic control and reduction of body weight in clinical trial [104]. Since then, similar drugs have been developed, and the Food and Drug Administration has approved Mounjaro (tirzepatide, a single molecule) that selectively binds to the receptors for both GIP and GLP-1 to treat T2DM [105]. However, there are acute side effects such as, qualmishness, loose stools, food aversion, and bloating abdomen.

Obesity is associated with insulin resistance. β3-adrenergic receptor (β3-AR), mainly located in urinary bladder, brown and white adipose tissues, has been shown to play an important role in activation of brown adipose tissue thermogenesis, white adipose tissue lipolysis, and insulin sensitivity [106,107]. Mirabegron, a β3-AR agonist that is only approved for the management of overactive bladder, activates thermogenesis of brown adipose tissue, lipolysis of white adipose tissue and insulin sensitivity in preclinical studies [108]. However, the experimental subjects are healthy women with normal BMI; further clinical trials on patients with metabolic diseases (i.e., diabetes and obesity) are warranted to confirm the efficacy of β3-AR agonist on the treatment of these diseases.

NAFLD is associated with obesity and is directly intertwined with insulin resistance and T2DM [109]. In varying degrees, experimental agents that target aspects of liver intermediary metabolism (such as ketohexokinase inhibitor, diacylglycerol acyltransferase inhibitor, PPARα-PPARδ agonist) have also proven beneficial, but their use may be limited by adverse effects [109]. Therefore, testing the approved and candidate drugs for diabetes and obesity in NAFLD patients would be promising.

Although new therapeutics for ameliorating insulin deficiency and insulin resistance have been developed for T2DM, most of the promising candidates were studied in experimental models and preclinical trials. For their final approval as clinical treatments, however, rigorous clinical trials with specific patients are required, especially factors such as age range, gender and ethnicity should be considered. In particular, biological sex plays a significant role in the development of metabolic diseases associated with insulin dysfunction, such as diabetes [110,111], cardiovascular diseases [112,113], and NAFLD [114–116]. The sexual hormone, estrogen, accounts in part for the fact that females are less susceptible than males. Estrogen therapy has been shown to improve cardiovascular health and insulin sensitivity [28,117,118], potentiating the critical role of estrogen in preserving metabolic health and insulin function. In the following section, we review the role of estrogen signaling in metabolic health and disease.

## Estrogen signaling

### Estrogen and its receptors

**Figure 2:**
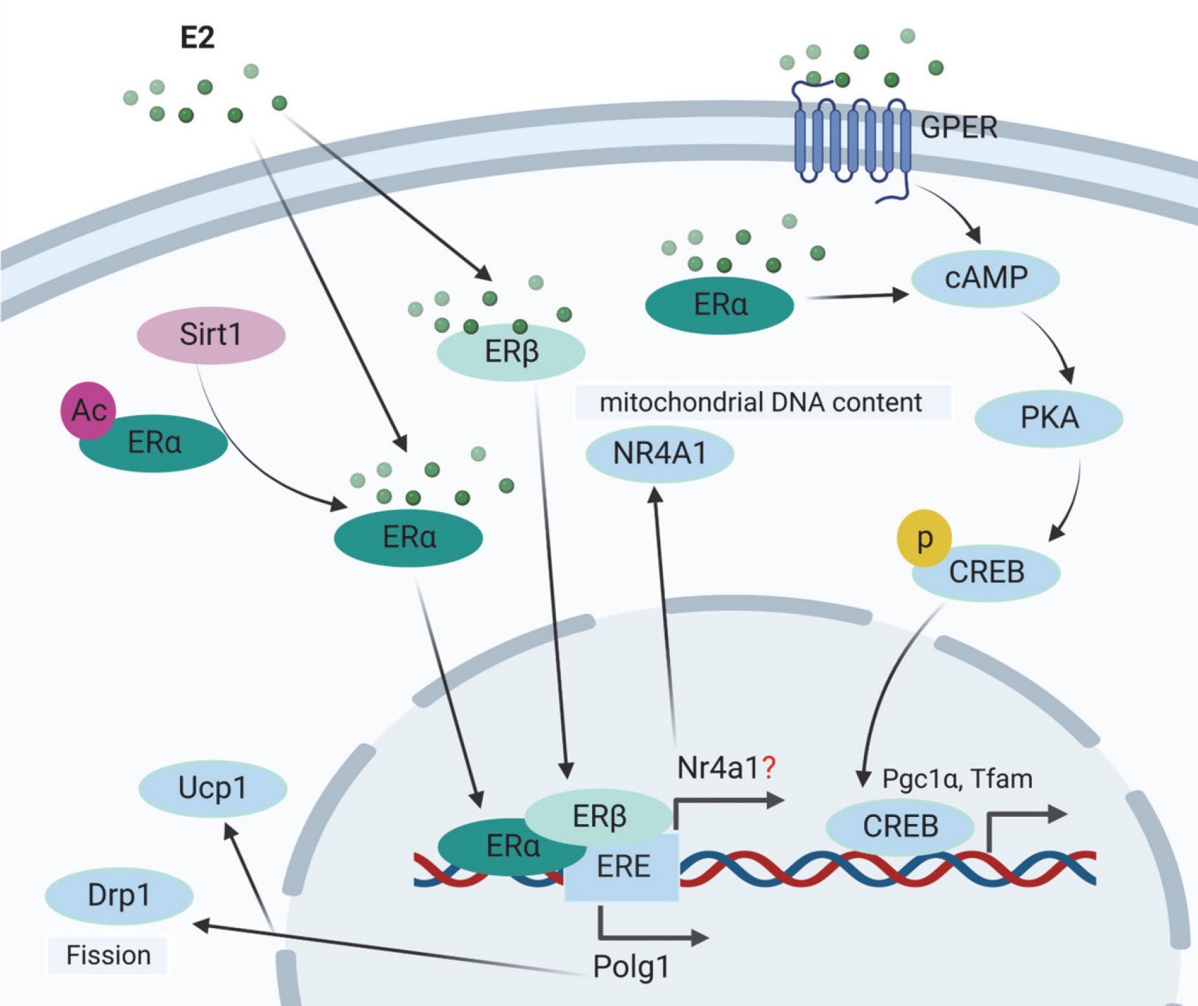
Estrogen signaling in metabolism and metabolic diseases Estrogen-ERα mediates mitochondrial metabolism through its receptors ERα and GPER. ERα increases cellular mitochondrial DNA content via nuclear receptor subfamily 4 group A member 1 (Nr4a1) activation. ERα mediates protein kinase A (PKA) dependent mitochondrial dynamic and mitophagy. In addition, ERα mediates mitochondrial fission by dynamin-related protein 1 (Drp1) and uncoupled respiration thermogenesis by uncoupled protein 1 (Ucp1), which is dependent on the transcriptional regulation of ERα on mtDNA polymerase γ subunit *Polg1*. GPER affects mitochondrial biogenesis through its activation of cAMP-PKA-CREB pathway.

Estrogen is a sex hormone that is involved in the development and regulation of the female reproductive system as well as secondary sex characteristics [119,120]. Estrone (E1), estradiol (E2), and estriol (E3) are the three primary endogenous estrogens with estrogenic hormonal activity, among which E2 is the most powerful and common one. The current review will focus on E2. Aromatase is the enzyme catalyzes the conversion of steroids to estrogens [17]. Estrogen is largely generated in the ovary in premenopausal women, while adipose tissue is the primary site for peripheral estrogen synthesis and metabolism [121,122]. Estrogen acts by binding to specific receptors, designated estrogen receptors (ERs), which activate transcriptional processes and/or signaling events that govern gene expression. There are three types of estrogen receptors, ERα, ERβ, and GPER (Figure 2A) [123,124]. Emerging evidence has indicated the critical functions of estrogen in maintaining metabolic health of extragonadal tissues, dysfunctional estrogen signaling has contributed to metabolic diseases, such as obesity, diabetes, cardiovascular disease, and liver disease [19,112,115,123,124].

The traditional process involves the cytosolic ERα being activated by the ligand (17b-estradiol, E2) coupled to heat shock protein 90 (Hsp90), dimerization of ERα, and direct DNA binding to estrogen response elements (ERE), or interaction with other transcription factors, such as the binding with AP1/SP1 sites [125]. Through ERα post-translational changes such as palmitoylation on active cystines and direct contact with caveolin-1, a tiny pool of ERα located near to the plasma membrane mediates the membrane-initiated signal (MISS) pathway. Membrane ERα interacts with protein kinases (Src and PI3K) or G-coupled protein ai (Gai) in response to E2 activation, activating endothelial NO synthase and several signaling cascades (Akt, PKA, and ERK1/2) (eNOS) [125]. Thus, estrogen signaling plays distinctive roles in metabolic diseases through different localization of ERs and may arise different signaling pathways.

### Estrogen-ER signaling in protein homeostasis

Estrogen signaling has been reported to affect protein stability and degradation, maintaining metabolic homeostasis, such as muscle strength and neuronal health [126–129]. Estrogens affect female skeletal muscle strength through mediating key proteins, protein synthesis and degradation, satellite cell function, apoptosis, and inflammation [126,127]. It has also shown that estrogen is essential for muscle recovery in female mice with Duchenne muscular dystrophy (DMD) [130]. Estrogens influence female skeletal muscle strength by preserving muscle mass and the quality of contractile proteins, such as myosin heavy chain and actin proteins [131,132]. Estrogens influence myosin heavy chain binding to actin to generate force via phosphorylation of the regulatory light chain. Protein turnover, proteolysis, and apoptosis are some of the processes influenced by estrogens that affect muscle mass [133–135]. Inflammation and satellite cell function are also estrogen sensitive and can contribute to muscle strength preservation [134,135]. Estrogen plays a crucial role in the processing of amyloid precursor protein (APP) and general neuronal health, which is partially via the modulation of brain-derived neurotrophic factor (BDNF), a factor that is similarly decreased in postmenopausal women and negatively associated with AD progression [128,129].

However, the direct target of estrogen has yet to be discovered. Although studies have implicated ERα in autophagic degradation pathway [136], the direct link between ERα, autophagy and these target degraded proteins should be elucidated.

### Estrogen-ER signaling in mitochondrial regulation

Estrogens affect mitochondrial functions, including their biogenesis, dynamics, and turnover through ERs. For instance, cardiomyocyte mitochondrial and plasma membrane localized ERα, not ERβ, controls mitochondrial structure and function [137]. However, ERβ has been found to be localized in cancer cells' mitochondria, where it appears to influence the action of mitochondrial DNA or mitochondrial transcription factors [138]. Constant ERα overexpression increases cellular ATP content as well as mitochondrial DNA content in differentiated myoblastic C2C12 cells via nuclear receptor subfamily 4 group A member 1 (Nr4a1) activation (Figure 2A); however, the direct regulation of ERα on Nr4a1 at the transcriptional level requires further investigation [139]. It has been shown that palmitic acid treatment in cells or high fat diet intervention in animals impaired hepatic mitochondrial function, inducing oxidative stress and activation of c-Jun N-terminal kinase (JNK) which has been related to the development of insulin resistance and steatosis [140]. E2 treatment induced mitochondrial biogenesis in line with decreased oxidative stress and suppression of sustained JNK activation [141].

ERα mediates mitochondrial dynamics and mitophagy, maintaining metabolic homeostasis and insulin sensitivity [23,142]. ERα in the muscle has been shown to be essential for systemic insulin sensitivity and lipid decomposition. Muscle specific ERα knockout impaired glucose homeostasis and increased adiposity via mediating protein kinase A (PKA) dependent mitochondrial dynamic and mitophagy (Figure 2A) [24]. Moreover, loss of ERα also confers to compromised mtDNA turnover by a balanced decrease in mtDNA replication and degradation [24]. In addition to ERα, GPER could also activate cAMP-PKA and mitochondrial function [143]. In 3T3L1 adipocytes, treatment of GPER agonist induces mitochondrial biogenesis, which was abrogated by PKA inhibitor, indicating the function of E2-GPER-PKA in mitochondrial biogenesis (Figure 2A) [143]. However, a recent study indicated that after postnatal development is complete, ERα function is no longer required to protect against HFD-induced skeletal muscle metabolic derangements. This may due to the duration of HFD treatment or mouse models [144]. In white adipose tissue, ERα controlled oxidative metabolism by restraining mitophagy. In brown adipose tissue, ERα mediates mitochondrial fission by dynamin-related protein 1 (Drp1) and uncoupled respiration thermogenesis by uncoupled protein 1 (Ucp1), which is dependent on the transcriptional regulation of ERα on mtDNA polymerase γ subunit *Polg1* (Figure 2A) [142]. The direct target of estrogen signaling and ERs in mitochondrial homeostasis is still under investigation.

### Impaired estrogen signaling and metabolic diseases

Biological sex plays a significant role in the development of metabolic diseases, such as diabetes [110,111], cardiovascular diseases [112,113], and NAFLD [114–116], with females experiencing more protection than males. For example, women have more body fat than males; however, the pear-shaped body fat distribution of many women is associated with decreased cardiometabolic risk, in contrast with the detrimental metabolic effects of apple-shaped obesity characteristic of men [145]. As it tends to diminish with the onset of menopause. For example, early menopause (before 40 years of age) or low levels of estrogens (also known as hypoestrogenaemia) in young women (18–40 years) both accelerated the morbidity of atherosclerosis [146,147], a 2.6-fold increase in CVD risk, and an increased risk of CVD-related mortality [148,149]. However, the loss of estrogen may be restored with hormone replacement treatment, this defense appears to be fueled by female sex hormones (estrogens) [18,111].

The critical roles of ERs among both genders in metabolic diseases have been underlined in both human and animal models. Clinical study indicated that the polymorphism of ERβ gene reduced the risk of developing T2D [150]. In physically active adults, polymorphism of ESR1 rs2234693 allele, not the T allele, protects against muscle injury by lowering muscle stiffness [151]. A systemic meta-analysis indicated the protective role of estrogen treatment on Alzheimer’s disease (AD) and Parkinson’s disease (PD) [152]. A clinical study indicated the beneficial role of estrogen on nigrostriatal dopaminergic neuron degeneration in PD [153]. Many of the functional implications for ERs in modulating cardiometabolic risk have been discovered in rodents, where female mice with ESR1 mutations develop age-dependent vascular dysfunction and metabolic syndrome traits such as obesity, glucose intolerance, and insulin resistance [154,155]. All the evidence underpins the critical role of ERs in maintaining metabolic homeostasis and potentiating derangement of estrogen signaling contributes to metabolic diseases.

Estrogen deficiency or loss of ERs has been collectively leads to metabolic syndrome. Both genetic and pharmacological manipulation of ERα and ERβ demonstrated the critical role of estrogen and receptors in maintaining metabolic diseases [125,156–158]. Global knockout ERα in mice recapitulate the phenotype of humans with rare inactivating receptor mutations and genetic polymorphisms in the receptor, which is exhibited by adiposity, reductions in energy expenditure and increased food intake, but they also exhibit glucose intolerance, insulin resistance, and reduced endothelial-derived nitric oxide production (vasculoprotective molecule), thus demonstrating the critical role for ESR1 in regulating energy, metabolic, and vascular homeostasis [155,159,160]. Estrogen deficiency in mice delayed myoregeneration in injured muscles and estrogen administration under ovariectomized conditions rescued delayed myoregeneration, suggesting that estrogen is an essential factor in the myoregeneration process via its receptor ERα and ERβ [161]. Specific muscle loss of ERα impaired muscle contractile function, implying that the beneficial effects of estradiol on muscle strength are dependent on ERα [162]. It has been revealed that ERα plays distinguishable role in sex-specific manner in liver diseases; however, Erα is essential for maintaining metabolic health in both genders [163,164]. For example, hepatic ERα plays a significant role in the sex-specific response to a HFD treatment and has different effects on the health of the liver in males and females as females are more susceptive to control the detrimental effects of a HFD than males, such as promoting mitochondrial fatty acid oxidation [164]. Despite less ERα protein expression in metabolically active tissues than female mice, male mice nonetheless need ERα for proper immunometabolic function, and ERα signaling is required for exercise-induced protection of hepatic steatosis as loss of ERα abolished the beneficial effects of physical exercise on immunometabolic function [163].

Given the critical role of estrogen and gender difference in metabolic disease, clinical trials should be designed to test drug efficacy and safety according to sex, age, and reproductive stage (i.e., menopause) as lack of adequate knowledge of gender difference and response in metabolic diseases [165,166]. For example, younger females are more susceptible to develop metabolic diseases than males due to difference in insulin sensitivity and glucose disposal ability (especially diabetes and its comorbidities) [167,168], while this tendency switches after the age of 40, potentiating the critical role of sexual hormones in controlling insulin signaling [169]. It may be due to the expression and active profile of ERs upon exercise in metabolically active tissues, such as muscle [170,171]. Therefore, understanding the molecular mechanism about how estrogen and ERs in maintaining metabolic homeostasis is critical for testing or designing new therapeutics for treating estrogen related metabolic diseases.

## The cross-talk of insulin with estrogen signaling

### Energy sensors Sirt1 and mTOR-mediated cross-talk

**Figure 3:**
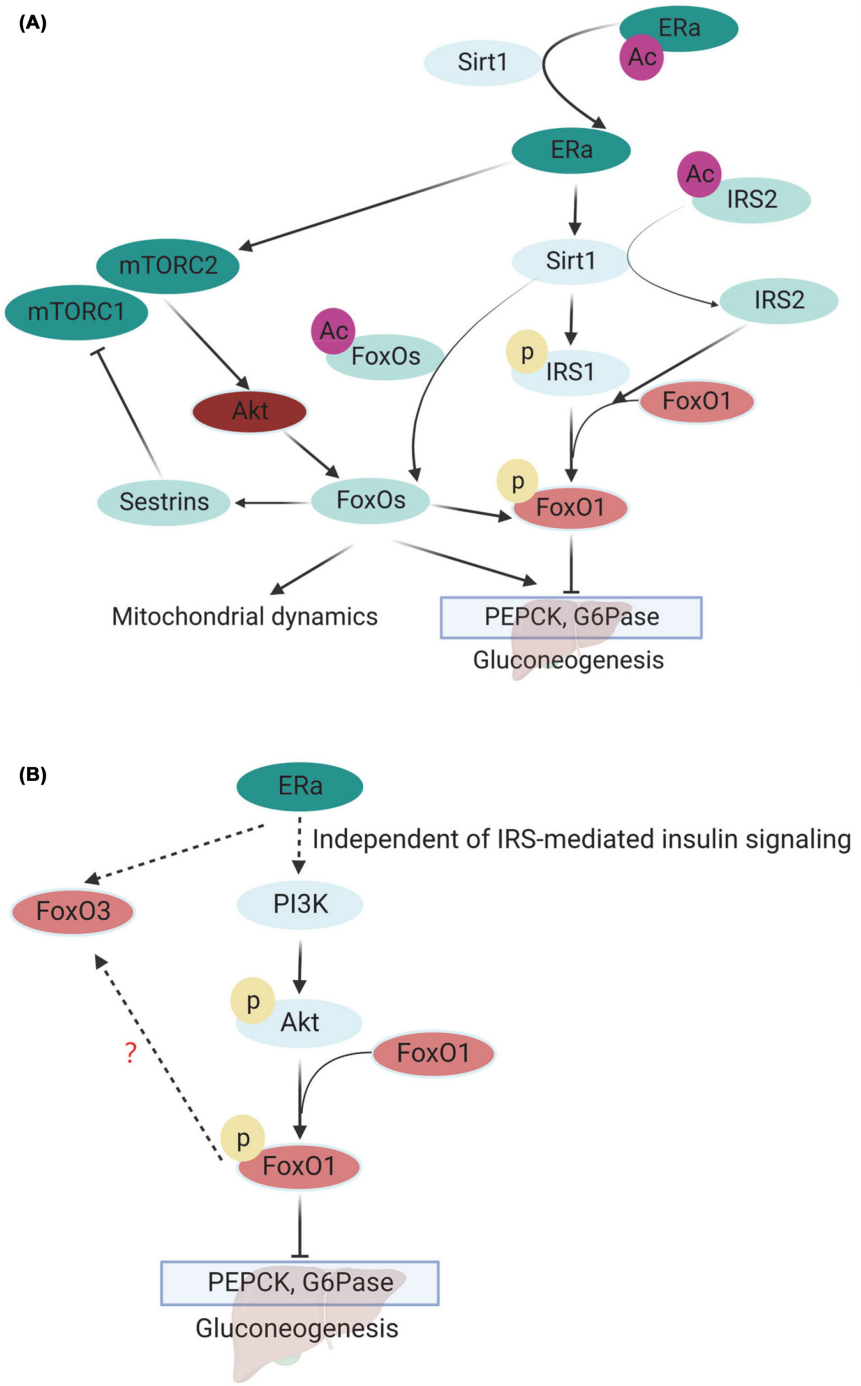
The cross-talk of insulin signaling with estrogen signaling (**A**) The convergence of insulin signaling and estrogen signaling via energy sensor Sirt1 or mTOR. ERα increases the level of Sirt1, which deacetylates IRS2 (multiple lysine sites) and regulates IRS1 phosphorylation, and it regulates downstream PI3K-Akt-FoxOs signaling. ERα interacts with FoxOs through the phosphorylation of mTORC2 on Akt (Ser473) and Akt (Thr308) on FoxOs, at the same time, FoxOs transcriptionally regulate Sestrins, which regulates mTOR signaling. (**B**) Estrogen- ERα cascade activates PI3K-Akt-FoxO1 independent of IRS-mediated insulin signaling and suppresses gluconeogenesis. Estrogen signaling activates FoxO3, which might be due to the compensatory role of FoxO3 for FoxO1 deactivation.

Insulin signaling controls nutrients metabolism and mitochondrial metabolism through targeting FoxOs and PGC1α (Figure 1A,B). Estrogen signaling fine-tunes protein turnover and mitochondrial metabolism primarily through its receptors, especially ERα (Figure 2A). Recently, Sirt1 has been found to interact with ERα, which converges insulin signaling and estrogen signaling. The interaction of ERα with Sirt1 is evident in the combination of deacetylation of ERα by Sirt1 and ERα transcriptional regulation of Sirt1 expression (Figure 3A) [26,27,172]. As a transcription factor, ERα binds to the promoter of Sirt1 and increases Sirt1 expression in breast cancer cell line [26]. We have for the first time revealed the cross-talk of ERα and Sirt1 in adipose tissue [27,173]. Our research reveals that a more active estradiol-ER signaling, which dials down autophagy and adipogenesis, is the cause of the lower visceral adiposity in females (compared with males) [136]. Furthermore, Sirt1 could function as downstream of ERα and also deacetylates ERα to inhibit mTOR-ULK1 dependent autophagy and adiposity [27,173]. This result revealed a new mechanism of Sirt1 regulating autophagy in adipocytes and shed light on sex difference in adiposity [27]. It has recently been discovered that E2 exerts its metabolic benefits by directly recruiting the ERα-Mc4r gene, which induces melanocortin-4 receptor (MC4R) signaling in the neurons of the ventromedial ventromedial hypothalamic nucleus (VMHvl) [174]. The regulatory fashion of ERs on other target genes like Sirt1 and Mc4r warrants further investigation.

Sirt1 could physically interacts with IRS2 and the deacetylase activity of Sirt1 is required for the deacetylation and tyrosine phosphorylation of IRS-2 upon insulin treatment, which is critical for the canonical insulin signaling (Figure 3A) [175,176]. In addition, Sirt1 promotes the expression of Rictor, a component of mTORC2, which phosphorylates Akt at Ser473, loss of Sirt1 in the liver confers to decreased expression of Rictor and decreased phosphorylation of Akt and constant activation of FoxO1 [177], which is opposite of the finding that liver specific loss of FoxO1 impairs fasting- and cAMP-induced glycogenolysis and gluconeogenesis [178]. Thus, how Sirt1 affects mTOR-Akt cascade should be further confirmed in different tissues and various physiological condition. Recently, we found that Sirt1 directly deacetylates Akt and increases its phosphorylation in adipocytes and mediates adipocyte autophagy and adiposity [27,179]. It provides evidence about how Sirt1 affects insulin signaling by direct targeting Akt in adipocytes. But how Sirt1 mediates insulin signaling in different cells and tissues under physiological and pathological conditions should be further explored. mTORC2 could phosphorylates Akt and activated Akt phosphorylates FoxOs and regulates downstream pathways. In addition, FoxOs could cross-talk with mTOR signaling through Sestrins (Figure 3A). Mechanistically, FoxO1 binds to the promoter of Sestrin3 and induces Sestrin3 expression, but not Sestrin1 and Sestrin2 [180,181], and Sestrin3 inhibits mTORC1 signaling in a TSC2-dependent manner. Although FoxOs and Sestrins have been extensively studied for their involvement in metabolic diseases [182–184]; however, how FoxOs-Sestrin-mTOR cascade participates in metabolic regulation and diseases is still largely unknown. It would be interesting to investigate this cascade affects cellular metabolism under different nutritional, hormonal, and pathological status.

### PI3K-mediated cross-talk

Estrogen–ER signaling has been found to maintain insulin sensitivity, potentiating the cross-talk of estrogen signaling and insulin signaling [28,185,186]. In male and ovariectomized (OVX) female mice, FoxO1 was found to be required for the improvement of estrogen on insulin resistance, which is through activation of ERα- PI3K-Akt-FoxO1 signaling, which is independent of IRS1 and IRS2 (Figure 3B) [28]. Moreover, subcutaneous 17β-estradiol (E2) implanting increased insulin sensitivity and decreased gluconeogenesis in male and ovariectomized (OVX) female control mice but not in liver-specific FoxO1 knockout (L-F1KO) animals. Activating the estrogen receptor ERα-PI3K-Akt-FoxO1 cascade, which is independent of classical insulin signaling involving IRS1 and IRS2, enables E2 to fine-tune glucose mentalism (Figure 3B) [28].

A similar study was shown in ovariectomized rats, subcutaneously administration of 17β-estradiol (E2) significantly improved insulin sensitivity in line with increased phosphorylation of Akt and its substrate Akt substrate of 160 kDa (AS160) Thr64, which was reported to regulate the plasma membrane-trafficking of GLUT4 [187,188]. However, a clinical study indicated the activation of FoxO3 by estrogen, acute E2 treatment potentiated the time-dependent effects of E2 on insulin by activating FoxO3, which is characterized by its dephosphorylation, and suppressing muscle atrophy in line with decreased MuRF1 protein level in early postmenopausal, but not late postmenopausal [189]. It might be due to the compensatory role of FoxO3 for FoxO1 deactivation. In addition, GSK3, as another substrate of PI3K-Akt, has been elucidated in ERα-Akt-GSK3 signaling pathway, in which estrogen prevents Tau phosphorylation, acting as a neuroprotective agent against the neurodegeneration of the female brain [190]. In addition, E2-induced and ERα-mediated glucose transporter 4 (GLUT4) translocation to plasma membrane is essential for glucose uptake [191,192], which also interacts with insulin signaling.

Furthermore, estrogen signaling may regulate insulin secretion by affecting pancreatic β cells. Treatment with estrogen restored insulin release from pancreatic β cells in mice genetically predisposed to protein misfolding due to insulin dysfunction, which is fine-tuned by activation of the endoplasmic-reticulum-associated protein degradation system [193,194]. Further studies are warranted to elucidate how estrogen medicates essential signaling pathways for preserving and restoring pancreatic insulin secretion. Estrogen signaling also affects other hormonal signaling pathways (e.g., FGF21). It has been shown that E2- β-cat/TCF-FGF21 signaling is essential for liver lipid metabolism and health; however, how ERα/ERβ are involved in β-cat/TCF binding to Fgf21 promoter and FGF21 is still not known, which warrants further investigation [25].

**Figure 4:**
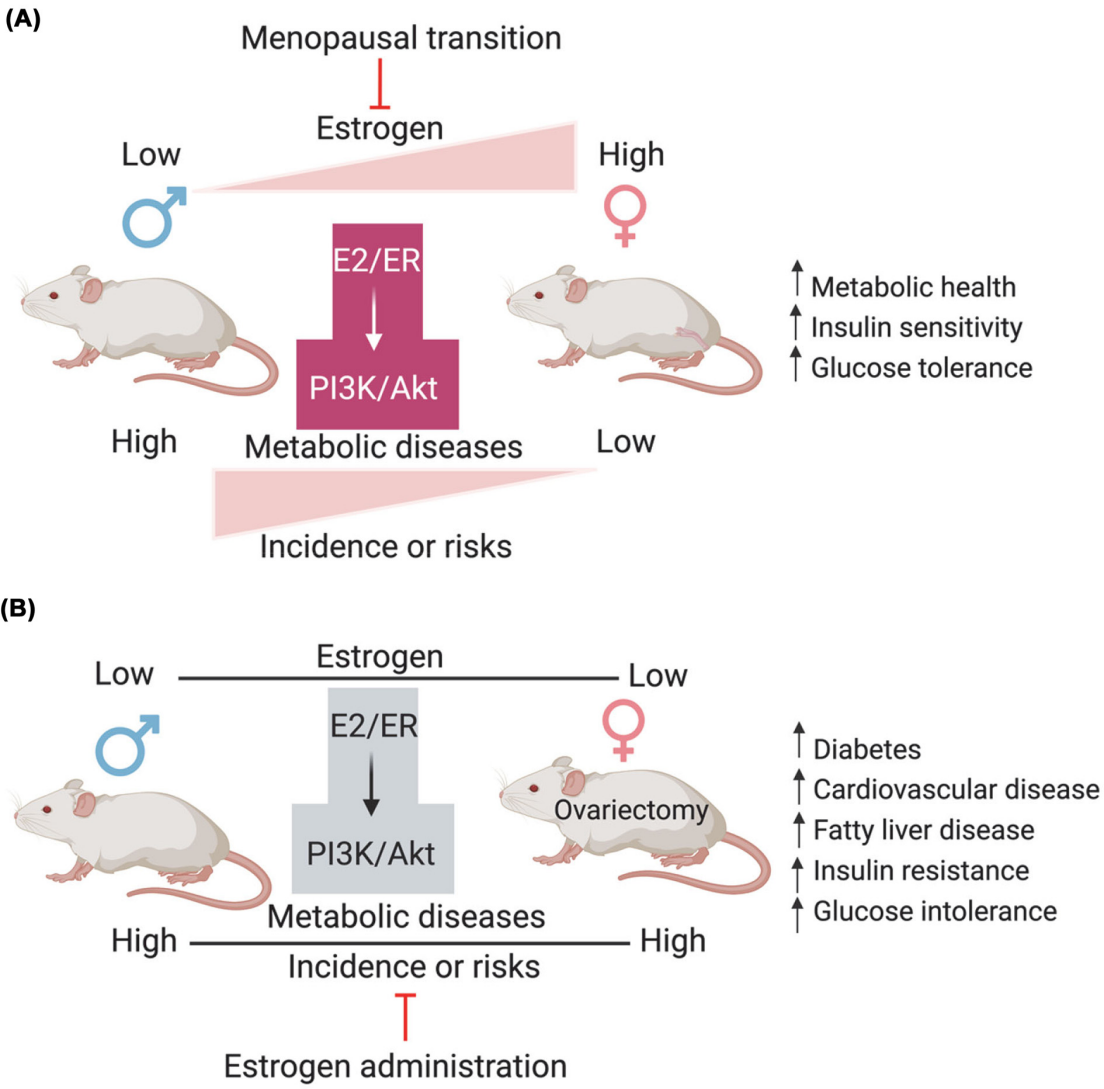
Sex difference in metabolic diseases and the role of estrogen in this sex-dependent regulation (**A**) In premenopausal conditions, females show lower risk of metabolic diseases and higher estrogen levels than males, mainly due to the activated PI3K-Akt pathway. The females exhibit metabolic health, insulin sensitivity, and glucose tolerance. However, the difference is normalized by menopausal transition, hallmarked by decreased estrogen secretion in females. (**B**) Ovariectomized (OVX) females exhibit metabolic diseases (such as diabetes, cardiovascular disease, and fatty liver disease), insulin resistance, and glucose intolerance. Administration of estrogen in males and OVX females improves metabolic health, insulin sensitivity and glucose tolerance, normalizing the sex difference. The potential target of estrogen is the activation of PI3K/Akt signaling.

Females at the age of premenopause show lower risk of metabolic diseases than age-matched males, which is mainly due to the physiological secretion and function of estrogen (Figure 4). However, after menopause, the sex difference is normalized due to reduced secretion of estrogen in females. Recent studies indicate that administration of estrogen improves insulin sensitivity and glucose tolerance in both male and OVX female mice [28], while this improvement is absent in liver-specific FoxO1 knockout (L-F1KO) mice, demonstrating the critical role of estrogen-FoxO1 axis in the protective effects. In both male and OVX females, heart specific IRS1/2 double knockout induces cardiac insulin resistance and diabetic cardiomyopathy [117]. Administration of estrogen improves the cardiac conditions and insulin sensitivity in both males and OVX females, suggesting that IRS1/2 is dispensable for the effects of estrogen, in line with activation of Akt and FoxO1 by estrogen [117]. Together, estrogen plays a critical role in the sex difference of metabolic diseases (Figure 4), in part by targeting on PI3K and the downstream Akt-FoxO1 signaling.

## Conclusion

Accumulated evidence depicts a great deal of insulin signaling and estrogen signaling in metabolic regulation. Dysregulation of insulin and estrogen signaling causes metabolic diseases, such as obesity, diabetes, cardiovascular diseases, muscle diseases, liver diseases, and neurodegenerative diseases. Although distinctive in pathways, insulin signaling and estrogen signaling may regulate macronutrients metabolism, mitochondrial homeostasis (mitochondrial biogenesis, dynamics and mitophagy) and other hormones. Insulin signaling affects macronutrients metabolism and mitochondrial metabolism mainly through IRS-PI3K-Akt regulation of FoxOs and PGC1α. Estrogen signaling fine-tunes protein turnover and mitochondrial metabolism through its receptors (ERα, ERβ and GPER), especially ERα. In addition, insulin signaling may interact with estrogen signaling via Sirt1, mTOR and PI3K. E2-ERα transcriptionally promotes Sirt1 expression and in turn Sirt1 could deacetylate ERα, which mediates mTOR signaling and autophagy. Sirt1 deacetylates FoxOs, which is the key transcription factor in insulin signaling. FoxOs interplays with mTOR signaling, in which FoxOs transcriptionally regulate Sestrins and Sestrins-mTOR signaling node. However, how FoxOs-Sestrins-mTOR cascade participates in the cellular metabolism and metabolic diseases remains largely unknown. On the other hand, insulin signaling interplays with estrogen signaling through PI3K. But how E2-ERα activates PI3K-Akt-FoxOs needs more investigations. Future studies are warranted to further elucidate the function of insulin signaling and estrogen signaling, especially their cross-talk in specific tissues and at global level, for the purpose of preventing dysfunctional hormone-related metabolic diseases and designing new therapeutic strategies.

## Data Availability

NA

## Open Access

Open access for this article was enabled by the participation of University of Florida in an all-inclusive *Read & Publish* agreement with Portland Press and the Biochemical Society under a transformative agreement with Individual.

## Abbreviations

β3-AR: β3-adrenergic receptor
AD: Alzheimer’s disease
Akt: Protein kinase B
AMPK: AMP-activated protein kinase
aPKCλ: atypical protein kinase C λ
APP: amyloid precursor protein
AS160: Akt substrate of 160 kDa
BDNF: brain-derived neurotrophic factor
c-Raf: RAF proto-oncogene serine/threonine-protein kinase
CBP: CREB-binding protein
CeRNA: Competing endogenous RNA
Clk2: Cdc2-like kinase 2
CREB: cAMP response element-binding protein
CRTC2: transcription coactivator 2
CVD: cardiovascular disease
DMD: Duchenne muscular dystrophy
Drp1: Dynamin-related protein 1
E2: estradiol
ERα: estrogen receptor α
ERβ: estrogen receptor β
*FGF21*: fibroblast growth factor 21
Fgf21: fibroblast growth factor 21 gene
FoxO1: Forkhead Box O1
FoxO3: Forkhead Box O3
FoxO4: Forkhead Box O4
G6Pase: glucose-6-phosphatase
Gai: G-coupled protein ai
GIP: glucose-dependent insulinotropic polypeptide
GLP1: glucagon-like peptide-1
GLUT1: glucose transporter 1
GLUT4: glucose transporter 4
GPER: G-protein-coupled estrogen receptor 1
GSIS: glucose-stimulated insulin secretion
GSK3: glycogen synthase kinase-3
HFD: high fat diet
Hsp90: Heat shock protein 90
IHTG: intrahepatic triglyceride
IR: insulin receptor
IRS1: insulin receptor substrate-1
IRS2: insulin receptor substrate-2
JNK: c-Jun N-terminal kinase
LncRNA: long non-coding RNA
LTKO^IRS^: liver-specific Irs1, Irs2 and FoxO1 triple knockout
MC4R: Melanocortin-4 receptor
MEK: mitogen-activated protein kinase
miR-484: microRNA-484
mTORC1: mammalian target of rapamycin complex 1
mTORC2: mammalian target of rapamycin complex 1
NAFLD: nonalcoholic fatty liver disease
NRF1: nuclear Respiratory Factor 1
OVX: ovariectomized
PAK: p21 (RAC1) activated kinase
PAK1: p21 (RAC1) activated kinase 1
PAK1/2: p21 (RAC1) activated kinase 1/2
PAK2: p21 (RAC1) activated kinase 2
PD: Parkinson’s disease
PDPK1: phosphoinositide-dependent kinase 1
PEPCK: phosphoenolpyruvate carboxykinase
PGC-1α: peroxisome proliferator-activated receptor γ coactivator 1-α
PI3K: phosphoinositide 3-kinase
SAT: subcutaneous adipose tissue
SIRT1: Sirtuin 1
STZ: streptozotocin
T1DM: Type 1 diabetes mellitus
T2DM: Type 2 diabetes mellitus
Tfam: transcription Factor A
Ucp1: uncoupled protein 1
VAT: visceral adipose tissue
VMHvl: ventromedial hypothalamic nucleus
